# The Sequential Organ Failure Assessment (SOFA) Score: has the time come for an update?

**DOI:** 10.1186/s13054-022-04290-9

**Published:** 2023-01-13

**Authors:** Rui Moreno, Andrew Rhodes, Lise Piquilloud, Glenn Hernandez, Jukka Takala, Hayley B. Gershengorn, Miguel Tavares, Craig M. Coopersmith, Sheila N. Myatra, Mervyn Singer, Ederlon Rezende, Hallie C. Prescott, Márcio Soares, Jean-François Timsit, Dylan W. de Lange, Christian Jung, Jan J. De Waele, Greg S. Martin, Charlotte Summers, Elie Azoulay, Tomoko Fujii, Anthony S. McLean, Jean-Louis Vincent

**Affiliations:** 1 Hospital de São José, Centro Hospitalar Universitário de Lisboa Central, Faculdade de Ciências Médicas de Lisboa, Nova Médical School, Lisbon, Portugal; 2 Faculdade de Ciências da Saúde, Universidade da Beira Interior, Covilhã, Portugal; 3 Adult Critical Care, St. George’s University Hospitals NHS Foundation Trust, St. George’s University of London, London, UK; 4 Adult Intensive Care Unit, Lausanne University Hospital and Lausanne University, Lausanne, Switzerland; 5 Departamento de Medicina Intensiva, Facultad de Medicina, Pontificia Universidad Católica de Chile, Santiago, Chile; 6 Department of Intensive Care Medicine, Bern University Hospital, University of Bern, Bern, Switzerland; 7 Division of Pulmonary, Critical Care, and Sleep Medicine, University of Miami Miller School of Medicine, Miami, FL USA; 8 Department of Anesthesiology, Critical Care, and Emergency Medicine, Hospital de Santo António - Centro Hospitalar Universitário Do Porto, Porto, Portugal; 9 Department of Surgery, Emory Critical Care Center, Atlanta, GA 30322 USA; 10 Department of Anaesthesiology, Critical Care and Pain, Tata Memorial Hospital, Homi Bhabha National Institute, Mumbai, Maharashtra India; 11 Division of Medicine, Bloomsbury Institute of Intensive Care Medicine, University College London, London, UK; 12 Hospital Do Servidor Público Estadual “Francisco Morato de Oliveira”, São Paulo, SP Brasil; 13 Department of Medicine, University of Michigan, Ann Arbor, MI USA; 14 Department of Critical Care, D’Or Institute for Research and Education (IDOR), Rio de Janeiro, Brazil; 15 Medical and Infectious Diseases Intensive Care Unit (MI2), AP-HP, Bichat Hospital, Paris, France; 16 Department of Intensive Care Medicine, University Medical Centre Utrecht, University Utrecht, Utrecht, The Netherlands; 17 Division of Cardiology, Pulmonology and Vascular Medicine, Medical Faculty, University Hospital Düsseldorf, Heinrich Heine University Düsseldorf, 40225 Düsseldorf, Germany; 18 Department of Critical Care Medicine, Ghent University Hospital, Ghent, Belgium; 19 Department of Medicine, Division of Pulmonary, Allergy, Critical Care and Sleep Medicine, Emory University and Grady Memorial Hospital, Atlanta, GA USA; 20 Heart and Lung Research Institute, University of Cambridge, Cambridge, UK; 21 Medical Intensive Care Unit, Famirea Study Group, Paris, France; 22 Intensive Care Unit, Jikei University Hospital, Tokyo, Japan; 23 Department of Intensive Care Medicine, Nepean Hospital, Kingswood, NSW Australia; 24 Department of Intensive Care, Erasme Hospital, Université Libre de Bruxelles, 1070 Brussels, Belgium; 25 VA Center for Clinical Management Research, HSR&D Center of Innovation, Ann Arbor, MI USA

## Abstract

The Sequential Organ Failure Assessment (SOFA) score was developed more than 25 years ago to provide a simple method of assessing and monitoring organ dysfunction in critically ill patients. Changes in clinical practice over the last few decades, with new interventions and a greater focus on non-invasive monitoring systems, mean it is time to update the SOFA score. As a first step in this process, we propose some possible new variables that could be included in a SOFA 2.0. By so doing, we hope to stimulate debate and discussion to move toward a new, properly validated score that will be fit for modern practice.

**Supplementary Information:**

The online version contains supplementary material available at 10.1186/s13054-022-04290-9.

## Background

The Sequential Organ Failure Assessment (SOFA) score was developed in 1994 at a Consensus Conference of the Working Group on Sepsis-Related Problems of the European Society of Intensive Care Medicine in Versailles and published in 1996 (Table 1) [1]. Although originally known as the "Sepsis-Related” Organ Failure Assessment score, the name was soon changed to “Sequential” Organ Failure Assessment as it is also applicable to critically ill patients without sepsis [2]. The SOFA score rapidly became one of the most widely used scoring systems in adult intensive care, both in clinical practice and research [3–5].

**Table 1:** Original Sequential Organ Failure Assessment (SOFA) score [2]

Score	0	1	2	3	4
*Respiratory*					
PaO_2_/FiO_2_, mmHg	> 400	≤ 400	≤ 300	≤ 200	≤ 100
				—with respiratory support—	
*Coagulation*					
Platelets × 10^3^/mm^3^	> 150	≤ 150	≤ 100	≤ 50	≤ 20
*Liver*					
Bilirubin, mg/dL (μmol/L)	< 1.2 (< 20)	1.2–1.9 (20–32)	2.0–5.9 (33–101)	6.0–11.9 (102–204)	> 12.0 (> 204)
*Cardiovascular*					
Hypotension	No hypotension	MAP < 70 mmHg	Dopamine ≤ 5 or dobutamine (any dose)*	Dopamine > 5 or epinephrine ≤ 0.1 or norepinephrine ≤ 0.1*	Dopamine > 15 or epinephrine > 0.1 or norepinephrine > 0.1*
*Central nervous system*					
Glasgow Coma Scale	15	13–14	10–12	6–9	< 6
*Renal*					
Creatinine, mg/dL (μmol/L)	< 1.2 (< 110)	1.2–1.9 (110–170)	2.0–3.4 (171–299)	3.5–4.9 (300–440)	> 5.0 (> 440)
OR urine output				< 500 ml/d	< 200 ml/d

^*^Adrenergic agents administered for at least one hour (doses given are in mcg/kg/min)

The score was designed to be easy to use and to fulfil a number of guiding principles [1]:

1. Organ dysfunction/failure is a process rather than an event so should not be seen simply as ‘present’ or ‘absent’ but rather as a continuum that can be objectively graded.
2. Because organ function can change very quickly in critically ill patients, it must be possible to repeat the score regularly (at least once a day) in order to describe a time course rather than the simple presence or absence of organ dysfunction/failure.
3. The number of variables should be kept low, making computation as simple as possible. The variables should be rapidly available and routinely obtained in every institution.

The primary purpose of the SOFA score is, as far as is possible, to objectively describe organ (dys)function rather than to predict outcome, so no associated equation was developed for mortality prediction. This is an important distinction from severity scores such as the Acute Physiology and Chronic Health Evaluation (APACHE) score or Simplified Acute Physiology Score (SAPS) that have a different purpose, i.e., to evaluate the risk of death at hospital discharge based on data collected at admission or during the first 24 h in the intensive care unit (ICU). It was also decided that the SOFA score should include sub-scores for the different organs considered, to permit evaluation of each organ individually, in addition to the global score.

Intensive care medicine has evolved considerably since the SOFA score was first proposed, with some interventions and management strategies abandoned or replaced, improved processes of care, and new procedures and treatments available. We believe it is therefore time to update the SOFA score to better reflect current practice.

In this brief perspective, our aim is not to create and validate a new score, but rather to highlight potential challenges and areas for ongoing deliberation in the development of a SOFA 2.0 score. We obtained an informal consensus among the authors, many of whom have decades of experience with the SOFA score and are thus aware of its strengths and weaknesses. Importantly, management of critically ill pediatric patients differs from that of adults, as do their physiological variables, so our discussions relate only to adult patients.

## Moving from SOFA 1.0 to SOFA 2.0?

We believe that when updating the SOFA score, the fundamental principles outlined above should be retained. The score should be kept as simple as possible by including a limited number of objective variables—acknowledging the presence of iatrogenic confounders, such as sedation for the Glasgow Coma Scale (GCS) score—which are easily obtained and routinely measured in every institution, and retaining the same 0–4 scale for each organ system.

Many (bio)markers of organ function have been studied since the initial SOFA score was developed but have not been extensively validated and are clearly not available everywhere. Some of the variables proposed in the original SOFA score may therefore still represent the most widely available and reliable indicator of function for that organ system albeit with acknowledged limitations. For example, bilirubin concentrations may still be the best choice for the hepatic system even though raised bilirubin can be due to hemolysis rather than liver dysfunction and hyperbilirubinemia takes time to develop. Similarly, although the platelet count can be normal despite an abnormal prothrombin or partial thromboplastin time and clearly does not provide a full picture of coagulopathy, it may still represent the best option for assessing function of the coagulation system.

Assessment of central nervous system function is particularly challenging given the lack of available objective measures. The GCS score, although subjective, remains an obvious choice given its relative simplicity and extensive validation. Nevertheless, the use of sedative agents makes its interpretation difficult in some patients, in particular those receiving mechanical ventilation. In these circumstances, an assumed GCS, i.e., the score that the patient would have in the absence of sedation, could be used, as is currently recommended [1], recognizing that this may not be compatible with the fully automated data collection systems that are increasingly employed [6].

As noted during the development of the original SOFA score [1], the variables selected for each organ should ideally be independent of therapy, as management practices vary across units and patients depending on availability and hospital and/or physician preference. However, for the cardiovascular and respiratory systems this may not be possible. If the current use of therapeutic agents for the cardiovascular system is retained in a SOFA 2.0, several changes in use of vasopressor and inotropic agents to primarily correct hypotension or cardiac output merit consideration for inclusion. For example, vasopressin and its derivatives are now used in many centers [7, 8]. Although less widely used, metaraminol, phenylephrine and angiotensin II are other vasopressors that could be considered for inclusion [3, 8, 9]. While use of dopamine has declined considerably worldwide, it may still be used sufficiently to warrant retention [10, 11]. Inclusion of other inotropic agents, such as levosimendan and phosphodiesterase (PDE)-3 inhibitors [12], may also be considered in addition to dobutamine. At which degree of severity these variables should be included and which doses/cutoffs should be used would need prospective validation. Use of venoarterial extracorporeal membrane oxygenation (VA-ECMO), cardiac assist devices, or other support systems may also be considered in the assessment of the cardiovascular system [13], although such support may impact on the evaluation of the function of other organ systems. For example, a patient with severe cardiogenic shock receiving VA-ECMO will often have a very high PaO_2_ (i.e., > 400). When considering these issues, it will be important to not over-complicate the score while, at the same time, being generic for the different approaches in current use.

Another variable that could be considered to quantify the severity of cardiovascular dysfunction is blood lactate concentration. This can be easily monitored, values are related to morbidity and mortality in almost every critically ill patient, and a decrease during initial resuscitation generally indicates a good response to treatment [14]. However, changes in lactate concentration are relatively slow and values may remain elevated after apparently adequate resuscitation. Moreover, concentrations may be raised by factors other than tissue hypoxia, for example, liver function and drugs [15], so their inclusion in a SOFA 2.0 would need prospective evaluation of utility.

A key change in clinical practice has been the gradual shift toward less invasive monitoring. Hence, for the respiratory system, the use of a PaO_2_ value obtained from blood gas analysis could potentially be replaced by SpO_2_ measured by pulse oximetry [16]. However, this value is an approximation, as SpO_2_ is subject to more bias than is PaO_2_ [17], especially in the absence of positive end-expiratory pressure (PEEP). If the SpO_2_/FiO_2_ ratio were to be included as an alternative to evaluate and score oxygenation, as recently recommended [18], a relatively complex mathematical conversion is necessary using nonlinear equations [19, 20]. Conversion tables are, however, available to simplify this process (see Additional file 1: Table S1).

The need for “respiratory support” is currently a criterion for a respiratory sub-SOFA score of 3 or 4, which could now include use of high-flow oxygen therapy (HFOT) [21], non-invasive mechanical ventilation, and even venovenous extracorporeal membrane oxygenation (VV-ECMO) [22, 23] as these are more widely used. Similarly, renal replacement therapies are now widely available and could be considered as an indicator of renal dysfunction, unless used for non-renal indications (e.g., removal of toxic products). Use of other organ support techniques, such as liver replacement therapies, may need to be considered in the future, but these remain experimental at present.

Addition of other organ systems, such as gastrointestinal, metabolic or immune, could be considered, but it is unclear which variables could be used at the bedside to objectively evaluate function. Indeed, the gut was considered in the initial SOFA score, but excluded for these reasons [1]. Moreover, the simplicity of SOFA is one of its key features; adding more organ systems would increase complexity and thus reduce its global accessibility.

We are fully aware that some of the variables in any scoring system may not be measured every day, especially in low resource countries. The variable that is most frequently missing from the current SOFA score is the bilirubin concentration [24, 25], usually because the clinician assumes the level is normal so does not measure it. This is in agreement with the general rule from the original score developers that missing values are considered as normal for calculation of the SOFA score. Other options for dealing with missing data are available and need to be considered when creating SOFA 2.0, particularly in an era with increased use of automated data abstraction.

## Conclusion

The SOFA score is now over 25 years old. Being able to objectively describe patterns of organ dysfunction in critically ill patients is as relevant now as ever. However, with changes in clinical practice over the years, some aspects of the SOFA score may no longer be as relevant as they once were. As noted in the original publication, *“…any given score is not established indefinitely. This is a continuing process, requiring regular re-evaluation*” [1]—perhaps now is the time for such re-evaluation.

In this perspective, we have suggested some additional elements that could be considered in a SOFA 2.0, taking into account the need to keep the score simple and available to all (Table 2). There may well be others that we have not mentioned. Our aim herein is to provide a starting position for a SOFA score update and raise discussion. It is our intent to progress next to data dive, ideally from varied healthcare settings, followed by a more formal Delphi-type consensus, and then external validation—ideally prospective but perhaps initially using existing datasets. Full validation of the cutoffs for the different scores for each organ/system would be needed before SOFA 2.0 could safely replace the original SOFA score.

**Table 2:** Some variables that may be considered for inclusion in a Sequential Organ Failure Assessment (SOFA) score version 2.0, while still keeping it simple and accessible to all

Organ system	Current measure	Possible additions/alternatives for consideration
Hepatic	Bilirubin concentration	Clinical assessment of jaundice
Coagulation	Platelet count	Platelet transfusion
Respiratory	PaO_2_/FiO_2_, respiratory support	SpO_2_, HFO, NIV, VV-ECMO
Cardiovascular	Hypotension, norepinephrine, dopamine, dobutamine, agents	Vasopressin (and derivatives), phenylephrine, metaraminol, angiotensin II, other inotropes, VA-ECMO, cardiac support devices, blood lactate
Central nervous system	(Assumed) GCS score	GCS after sedation hold
Renal	Creatinine, urine output	RRT

*RRT* renal replacement therapy; *VA-ECMO* venoarterial extracorporeal membrane oxygenation; *VV-ECMO* venovenous extracorporeal membrane oxygenation; *GCS* Glasgow Coma Scale; *HFO* high-flow oxygenation; *NIV* noninvasive ventilation

## Supplementary Information

**Additional file 1**. Lookup table for imputed PaO_2_ for a given SpO_2_.

## Data Availability

Not applicable.

## Abbreviations

FiO2: Fraction of inspired oxygen
HFOT: High-flow oxygen therapy
IMV: Invasive mechanical ventilation
NIV: Noninvasive ventilation
PEEP: Positive end-expiratory pressure
RRT: Renal replacement therapy
SOFA: Sequential Organ Failure Assessment
VA-ECMO: Venoarterial extracorporeal membrane oxygenation
VV-ECMO: Venovenous extracorporeal membrane oxygenation
