# Supplementary material for: The Sequential Organ Failure Assessment (SOFA) Score: has the time come for an update?
Source: Crit Care. 2023 Jan 13;27:15. doi: 10.1186/s13054-022-04290-9 (PMC9837980; doi:10.1186/s13054-022-04290-9)
Supplement: Supplementary file 1 — Additional file 1. Lookup table for imputed PaO2 for a given SpO2. [file 13054_2022_4290_MOESM1_ESM.pdf]

Sequential Organ Failure Assessment (SOFA) Score – Has the Time Come For an Update?

Moreno et al

Electronic supplementary material

Table S1. Lookup table for imputed PaO2 for a given SpO2 based on non-linear equation (modified from [1])

Measured SpO2 (%)	Imputed PaO2 (mmHg)
100*	167**
99*	132*
98*	104*
97*	91*
96	82
95	76
94	71
93	67
92	64
91	61
90	59
89	57
88	55
87	53
86	51
85	50
84	49
83	47
82	46
81	45
80	44
79	43
78	42
77	42
76	41
75	40
74	39
73	39
72	38
71	37
70	37

*Generally considered unreliable on the basis of the sigmoidal shape of the hemoglobin-oxygen dissociation curve
**Based on SpO2 99.5%.

Reference
1. Brown SM, Duggal A, Hou PC, Tidswell M, Khan A, Exline M et al. Nonlinear imputation of PaO2/FIO2 from SpO2/FIO2 among mechanically ventilated patients in the ICU: A prospective, observational study. Crit Care Med. 2017;45:1317-24
